# Pharmacological Assessment of the Antiprotozoal Activity, Cytotoxicity and Genotoxicity of Medicinal Plants Used in the Treatment of Malaria in the Greater Mpigi Region in Uganda

**DOI:** 10.3389/fphar.2021.678535

**Published:** 2021-06-30

**Authors:** Fabien Schultz, Ogechi Favour Osuji, Anh Nguyen, Godwin Anywar, John R. Scheel, Guy Caljon, Luc Pieters, Leif-Alexander Garbe

**Affiliations:** ^1^ Institute of Biotechnology, Faculty III—Process Sciences, Technical University of Berlin, Berlin, Germany; ^2^ Department of Agriculture and Food Sciences, Neubrandenburg University of Applied Sciences, Neubrandenburg, Germany; ^3^ Department of Plant Sciences, Microbiology and Biotechnology, Makerere University, Kampala, Uganda; ^4^ Department of Global Health, University of Washington, Seattle, WA, United States; ^5^ Department of Radiology, University of Washington, Seattle, WA, United States; ^6^ Laboratory of Microbiology, Parasitology and Hygiene, Faculty of Pharmaceutical, Biomedical and Veterinary Sciences, University of Antwerp, Antwerp, Belgium; ^7^ Natural Products & Food Research and Analysis (NatuRA), Department of Pharmaceutical Sciences, University of Antwerp, Antwerp, Belgium; ^8^ ZELT—Neubrandenburg Center for Nutrition and Food Technology gGmbH, Neubrandenburg, Germany

**Keywords:** malaria, antiprotozoal, cytotoxicity, genotoxicity, medicinal plants, Uganda, Mpigi, ethnopharmacology

## Abstract

We investigated the potential antimalarial and toxicological effects of 16 medicinal plants frequently used by traditional healers to treat malaria, fever, and related disorders in the Greater Mpigi region in Uganda. Species studied were *Albizia coriaria*, *Cassine buchananii*, *Combretum molle*, *Erythrina abyssinica*, *Ficus saussureana*, *Harungana madagascariensis*, *Leucas calostachys*, *Microgramma lycopodioides*, *Morella kandtiana*, *Plectranthus hadiensis*, *Securidaca longipedunculata*, *Sesamum calycinum subsp. angustifolium*, *Solanum aculeastrum*, *Toddalia asiatica*, *Warburgia ugandensis,* and *Zanthoxylum chalybeum*. In addition, the traditional healers indicated that *P. hadiensis* is used as a ritual plant to boost fertility and prepare young women and teenagers for motherhood in some Ugandan communities where a high incidence of rapidly growing large breast masses in young female patients was observed (not necessarily breast cancer). We present results from various *in vitro* experiments performed with 56 different plant extracts, namely, 1) an initial assessment of the 16 species regarding their traditional use in the treatment of malaria by identifying promising plant extract candidates using a heme biocrystallization inhibition library screen; 2) follow-up investigations of antiprotozoal effects of the most bioactive crude extracts against chloroquine-resistant *P. falciparum* K1; 3) a cytotoxicity counterscreen against human MRC-5_SV2_ lung fibroblasts; 4) a genotoxicity evaluation of the extract library without and with metabolic bioactivation with human S9 liver fraction; and 5) an assessment of the mutagenicity of the ritual plant *P. hadiensis*. A total of seven extracts from five plant species were selected for antiplasmodial follow-up investigations based on their hemozoin formation inhibition activity in the heme biocrystallization assay. Among other extracts, an ethyl acetate extract of *L. calostachys* leaves exhibited antiplasmodial activity against *P. falciparum* K1 (IC_50_ value: 5.7 µg/ml), which was further characterized with a selectivity index of 2.6 (CC_50_ value: 14.7 µg/ml). The experiments for assessment of potential procarcinogenic properties of plant extracts *via* evaluation of *in vitro* mutagenicity and genotoxicity indicated that few extracts cause mutations. The species *T. asiatica* showed the most significant genotoxic effects on both bacterial test strains (without metabolic bioactivation at a concentration of 500 µg/plate). However, none of the mutagenic extracts from the experiments without metabolic bioactivation retained their genotoxic activity after metabolic bioactivation of the plant extract library through pre-incubation with human S9 liver fraction. While this study did not show that *P. hadiensis* has genotoxic properties, it did provide early stage support for the therapeutic use of the medicinal plants from the Greater Mpigi region.

## Introduction

In recent years, the prevalence of malaria infection and the incidence of related clinical disease has significantly decreased in sub-Saharan Africa (Bhatt et al., 2015; Snow et al., 2017). Nevertheless, malaria continues to be one of the most severe public health problems worldwide (CDC, 2021). In 2019, there were an estimated 229 million cases in 89 malaria-endemic countries associated with 409,000 deaths. Africa alone accounted for 94% of these cases (215 million) with 384,000 deaths (WHO, 2020). Human malaria is caused by five species of protozoan parasites of the genus *Plasmodium* that are transmitted through the bites of infected female *Anopheles* mosquitoes (Scholar, 2007; Kotepui et al., 2020). In Africa, 94% of malaria cases result from infection with the species *Plasmodium falciparum*, which poses the greatest malaria threat globally (WHO, 2020; CDC, 2021). Children under five years of age remain the most vulnerable group affected with 274,000 malaria deaths worldwide in 2019. In addition, in moderate to high transmission countries in the WHO African Region, malaria infection during pregnancy resulted in 822,000 children with low birthweight (Park et al., 2020; WHO, 2020).

**FIGURE 1:**
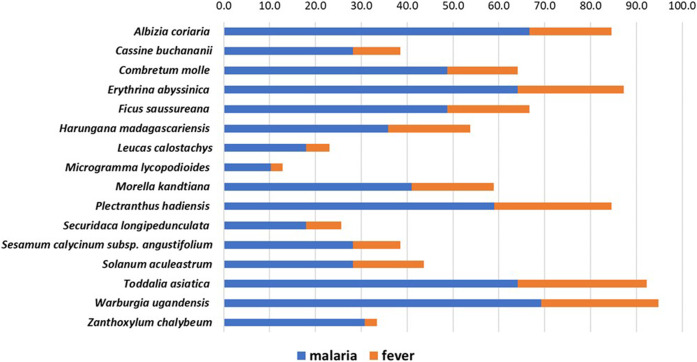
Stacked histogram for ethnopharmacological information from the Greater Mpigi region in Uganda, describing the medicinal use of 16 plant candidates (with emphasis on treatment of malaria and fever). The figure shows the relative frequencies of citation (RFCs) in %, a field assessment index, which was calculated from data obtained through an ethnobotanical survey of 39 traditional healers (Schultz et al., 2020b). Individual RFCs indicate the importance of each plant species used for treatment relative to the total number of informants interviewed in the study (*n* = 39). RFCs vary from 0% (none of the survey participants uses this plant species to treat a specific medical condition) to 100% (all survey participants use this plant species to treat a specific medical condition). Consequently, the higher the cumulative RFC values (*x*-axis), the more common the traditional use of a plant species in treatment of medical conditions caused by malaria infection.

In 2019, about 5% of the world’s malaria cases were recorded in Uganda alone (WHO, 2020). At our field study sites in the tropical Ugandan Greater Mpigi region and around Nakawuka village in Wakiso district, traditional medicine and the use of plants are still the predominant form of primary healthcare. In a recent study, we documented for the first time the traditional use of 16 medicinal plant species that are frequently used in local traditional medical practices (Schultz et al., 2020b). Figure 1 lists these species and their traditional use in treatment of malaria and fever, reporting the cumulated relative frequencies of citation in % (n = 39), which serve as the ethnopharmacological basis for this study.

When applying the novel Degrees of Publication (DoP) method for identification of species that merit the costly lab studies in ethnopharmacological research, the majority of these 16 species from the Greater Mpigi region were classified as being either highly understudied or understudied (Schultz et al., 2021a). Therefore, the present study further investigated the selected species for antimalarial activity and toxicity, contributing to an evaluation of traditional use and to drug discovery.

In this study, extracts of the 16 medicinal plants were initially investigated using a heme biocrystallization assay as a prescreen prior to antiplasmodial experiments with parasite cultures. The assay is based on monitoring the formation of the distinctive molecule hemozoin, which is a byproduct of the digestion of hemoglobin by the plasmodium parasite (Hempelmann, 2007). During infection and in the intraerythrocytic stage, the parasite metabolizes host hemoglobin within its digestive vacuole to facilitate parasite growth and development (Kapishnikov et al., 2019). This process involves the oxidation of hemoglobin to methemoglobin and subsequent hydrolysis by aspartic proteases to form denatured globin and free heme (Fe^3+^) (ferriprotoporphyrin IX). Free heme is toxic to the parasite and large quantities accumulate, reaching high concentrations that cause membrane disruption, lipid peroxidation, and protein and DNA oxidation. To prevent this, the free heme is detoxified by biocrystallization to form inert, insoluble hemozoin in the parasitic digestive vacuole (Schmitt et al., 1993; Becker et al., 2004; Kumar and Bandyopadhyay, 2005; Kumar et al., 2007; Coronado et al., 2014; Roy, 2017; Herraiz et al., 2019). The resulting hemozoin (biocrystallized heme) is a dark pigment that represents the characteristic black crystalline spot observed in red blood cells during diagnosis of patients infected with malaria (Egan, 2002; Combrinck et al., 2013; Coronado et al., 2014; Gupta et al., 2017). Thus, sequestration of heme into hemozoin is essential for the survival of *P. falciparum*, and the vital pathway of hemozoin pigment formation is one of the main targets of antimalarial drugs (Hempelmann, 2007; Herraiz et al., 2019; Kapishnikov et al., 2019). The mechanism of action of many antimalarial drugs on the market, such as quinacrine, amodiaquine, or chloroquine, is based on disruption of the heme detoxification pathway within the parasite, making these drugs potent heme biocrystallization inhibitors (Coronado et al., 2014; Herraiz et al., 2019). Interestingly, the mechanisms underlying hemozoin formation are yet to be fully elucidated and are poorly understood (Chugh et al., 2013; Herraiz et al., 2019).

There are a few reports of plants that, following long-term ingestion, are suspected to promote formation of breast tumors through mutagenesis and/or growth stimulation by phytoestrogens (Stopper et al., 2005; Bilal et al., 2014). The incidence rates of breast cancer are rapidly increasing in sub-Saharan Africa, including Uganda (Azubuike et al., 2018). Anecdotal reports from health providers at one of our other study sites, around Nakawuka village in Wakiso District, indicated a relatively high prevalence of rapidly enlarging breast masses in young women in these small rural communities (not necessarily cancer). Further discussions with local traditional healers and some affected women suggested that the medicinal plant *Plectranthus hadiensis* might be responsible. *P. hadiensis* (synonym: *P. cyaneus*), a member of the Lamiaceae family, is used regularly by local teenagers and young women as a ritual and medicinal plant to boost fertility and to “prepare women for marriage”, beginning with their first menstruation. The leaves of the plant are dried in the shade and subsequently pounded in a traditional mortar into powder. The women then mix the powder with Vaseline and apply it around the labia and administer intravaginally. Because of this frequent and long-term exposure to the plant and the potential connection to the undiagnosed rapidly growing large breast masses in women from the Nakawuka village area, we sought to be the first to preliminarily investigate potential genotoxic effects derived from *P. hadiensis*. Due to inefficiencies in the healthcare structure and limited social-economic abilities, access to non-traditional medical facilities for breast cancer detection and treatment is scarce and often unaffordable to the rural Ugandan population (Foerster et al., 2019; Nakaganda et al., 2021). The development of the mammary glands takes place in lifecycle windows that were previously identified as “hot-spots” for breast cancer risk (Martinson et al., 2013). Since the breasts are not fully developed when the first menstruation occurs and puberty involves a massive proliferation of cells, this window of susceptibility makes teenagers and young women particularly vulnerable for DNA damage caused by mutagens/carcinogens (Davis and Lin, 2011; Macias and Hinck, 2012; Natarajan et al., 2020). The administration of *P. hadiensis* and its genotoxic phytoestrogens could potentially serve as the first incident of cell growth perturbation that, in the long term, allows for further incidents to give rise to tumor growth and breast cancer.

**FIGURE 2:**
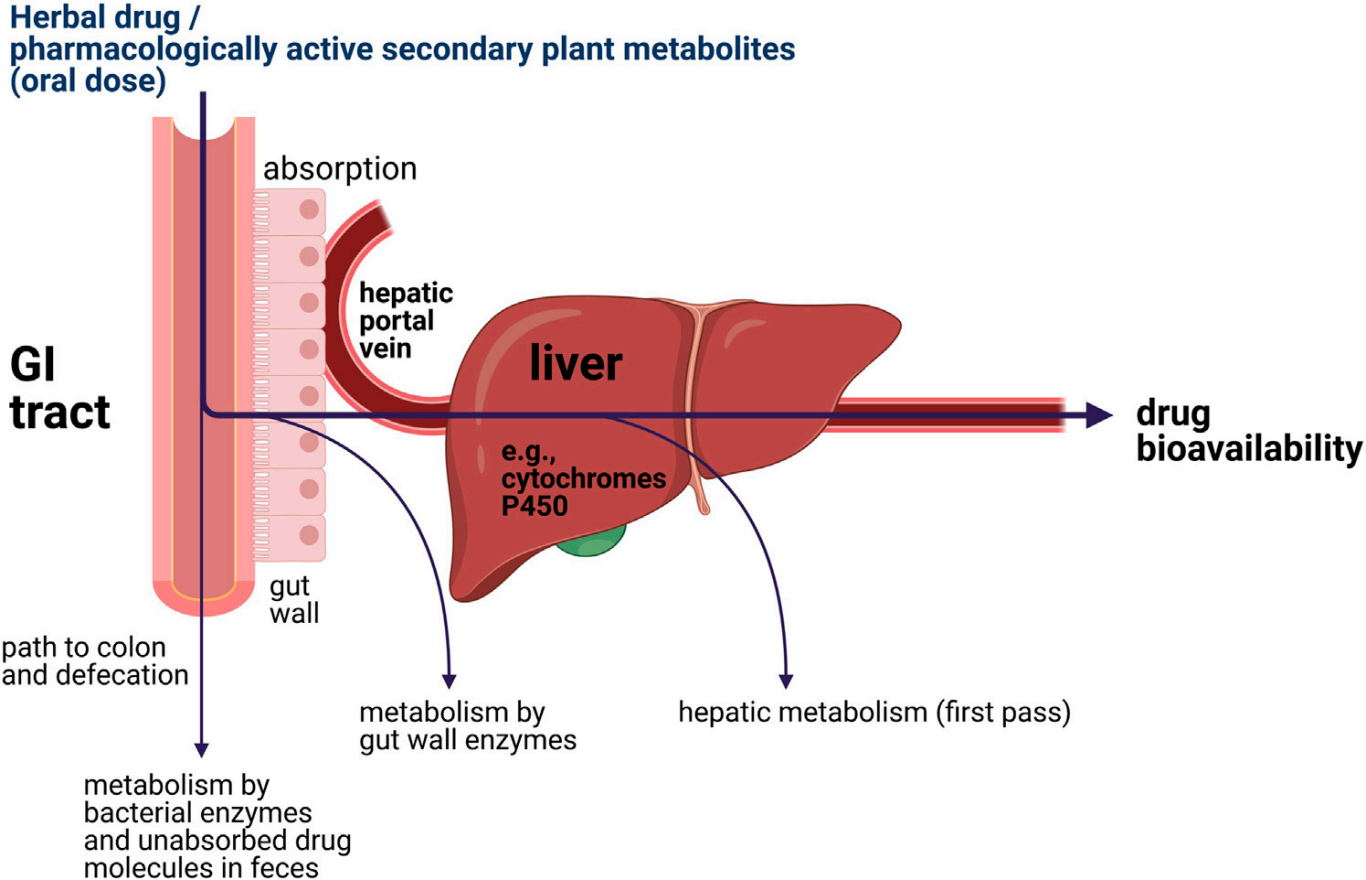
Simplified illustration of the locations of metabolic elimination of xenobiotics inside the human body.

Upon oral administration of a (herbal) drug, the human body will eventually try to eliminate xenobiotics. This often requires initial biotransformation for many drugs, which takes place in part in the gut wall but primarily in the liver (van de Waterbeemd and Gifford, 2003; Stavropoulou et al., 2018). This process is further illustrated in Figure 2. After oral intake, bioavailability of a drug or pharmacologically active secondary plant metabolites is dependent on two bioprocesses: absorption and metabolism (Rein et al., 2013; Chow, 2014). The oral bioavailability of a (herbal) drug is determined by the drug fraction of the initial dose that 1) was successfully absorbed by the gut wall; 2) appeared in the hepatic portal vein; and 3) ultimately entered the blood circulation after first pass through the liver metabolism (van de Waterbeemd and Gifford, 2003). It is important to note that the most essential liver enzymes for bioactivation of procarcinogens and biotransformation of (herbal) drugs are the cytochrome P450 enzymes, which oxidize substances using iron and are linked to a variety of reactions, including epoxidation, hydroxylation, S-oxidation, and O-dealkylation (Werck-Reichhart and Feyereisen, 2000; van de Waterbeemd and Gifford, 2003; Stavropoulou et al., 2018).

In this study, we investigate a unique library, composed of 56 extracts derived from the 16 Ugandan plant species, for their pharmacological *in vitro* activity. The objectives of this study were performing 1) an initial assessment of the 16 species regarding their traditional use in the treatment of malaria by identifying promising plant extract candidates using a heme biocrystallization inhibition library screen; 2) follow-up investigations of antiprotozoal effects of the most bioactive crude extracts against chloroquine-resistant *P. falciparum* K1; 3) a cytotoxicity counterscreen against human MRC-5_SV2_ lung fibroblasts; 4) a genotoxicity evaluation of the extract library; and 5) an assessment of mutagenicity of the ritual plant *P. hadiensis* that might be connected to an increased prevalence of breast cancer in young female patients in Eastern Uganda.

## Results

### Plant Species and Extractions

For each plant species, multiple extraction strategies were applied in order to look not only into the chemistry yielded by traditional preparation but also to investigate chemistries only accessible by alternative extraction methods and extractants. Consequently, plant extracts were regarded as chemical libraries in this study. The extraction methods were 1) maceration in either methanol, ethanol, ethyl acetate, or diethyl ether; 2) Soxhlet extraction using *n*-hexane and successively methanol; and 3) aqueous decoction conforming to the traditional method of preparation. The extractions yielded a total of 56 different plant extracts from 16 medicinal plant species. Taxonomic information on the species studied, local plant names in the Luganda language, sample identification numbers (extract IDs), plant parts selected for investigation, extraction solvents used, and herbarium voucher specimen numbers and locations are reported in Table 1.

**TABLE 1:** Information on collected plant species and different extracts investigated in the study.

Scientific name	Extraction solvent	Extract ID	Family	Local name in Luganda	Plant part	Voucher specimen # and location
*Securidaca longipedunculata* Fresen.	Ethyl acetate^a^	eE001	Polygalaceae	Mukondwe	Stem	AG196 (Makerere University Herbarium, Uganda)
	Water^a^	wE001				
	*n*-hexane (sox.)^a^	hE001				
	Methanol^a^	mE001				
	Methanol (sox. succ.)^a^	smE001				
*Microgramma lycopodioides* (L.) Copel.	Ethyl acetate^a^	eE002	Polypodiaceae	Kukumba	Root (rhizomes)	AG639 (Makerere University Herbarium, Uganda)
	Water^a^	wE002				
	Methanol^a^ (sox. succ.)	smE002				
*Ficus saussureana* DC.	Ethyl acetate^a^	eE003	Moraceae	Muwo	Stem	AG219 (Makerere University Herbarium, Uganda)
	Water^a^	wE003				
	*n*-hexane (sox.)^a^	hE003				
	Methanol^a^	mE003				
	Methanol (sox. succ.)^a^	smE003				
*Sesamum calycinum* subsp. *angustifolium* (Oliv.) Ihlenf. & Seidenst.	Ethyl acetate^a^	eE004	Pedaliaceae	Lutungotungo	Leaves	AG205 (Makerere University Herbarium, Uganda) 23173^*^ (Emory University Herbarium, United States of America)
	Water^a^	wE004				
	*n*-hexane (sox.)^a^	hE004				
	Methanol^a^	mE004				
	Methanol (sox. succ.)^a^	smE004				
*Leucas calostachys* Oliv.	Ethyl acetate^a^	eE005	Lamiaceae	Kakuba musulo	Leaves	AG195 (Makerere University Herbarium, Uganda) 23175^*^ (Emory University Herbarium, United States of America)
	Water^a^	wE005				
	*n*-hexane (sox.)^a^	hE005				
	Methanol (sox. succ.)^a^	smE005				
*Solanum aculeastrum* Dunal	Ethyl acetate^a^	eE006	Solanaceae	Kitengo	Root	AG193 (Makerere University Herbarium, Uganda)
	Water^a^	wE006				
	*n*-hexane (sox.)^a^	hE006				
	Methanol (sox. succ.)^a^	smE006				
*Albizia coriaria* Oliv.	Ethyl acetate^b^	eE007	Fabaceae	Mugavu	Stem bark	AG203 (Makerere University Herbarium, Uganda)
	Ethanol^b^	etE007				
*Erythrina abyssinica* DC.	Ethyl acetate^b^	eE008	Fabaceae	Jjirikiti	Stem bark	AG199 (Makerere University Herbarium, Uganda)
	Ethanol^b^	etE008				
*Zanthoxylum chalybeum* Engl.	Ethyl acetate^b^	eE009	Rutaceae	Ntaleyaddungu	Stem bark	AG204 (Makerere University Herbarium, Uganda)
	Ethanol^b^	etE009				
	Ethanol^c^	etE017				
	Diethyl ether^c^	dietE017				
	Ethanol^d^	etE017a				
	Diethyl ether^d^	dietE017a				
*Toddalia asiatica* (L.) Lam.	Ethyl acetate^c^	eE010	Rutaceae	Kawule	Leaves (80%)Bark (20%)	AG190 (Makerere University Herbarium, Uganda)
	Ethanol^b^	etE010				
	Diethyl ether^d^	dietE010				
	Ethanol^d^	etE010a				
*Harungana madagascariensis* Lam. ex Poir.	Ethyl acetate^d^	eE011	Hypericaceae	Mukabiiransiko	Stem bark	AG230 (Makerere University Herbarium, Uganda) 23180^*^ (Emory University Herbarium, United States of America)
	Ethanol^b^	etE011				
	Diethyl ether^d^	dietE011				
	Ethanol^d^	etE011a				
*Morella kandtiana* (Engl.) Verdc. & Polhill	Ethanol^b^	etE012	Myricaeae	Mukikimbo	Root	AG201 (Makerere University Herbarium, Uganda) 23174^*^ (Emory University Herbarium, United States of America)
	Ethanol^d^	etE012a				
	Diethyl ether^d^	dietE012				
*Cassine buchananii* Loes.	Ethyl acetate^b^	eE013	Celastraceae	Mbaluka	Stem bark	AG198 (Makerere University Herbarium, Uganda)
	Ethanol^b^	etE013				
	Ethanol^d^	etE013a				
*Warburgia ugandensis* Sprague	Ethanol^d^	etE014a	Canellaceae	Abasi	Stem bark	AG220 (Makerere University Herbarium, Uganda) 23181^*^ (Emory University Herbarium, United States of America)
	Diethyl ether^d^	dietE014				
*Combretum molle* R.Br. ex G.Don	Ethyl acetate^b^	eE015	Combretaceae	Ndagi	Stem bark	AG191 (Makerere University Herbarium, Uganda)
	Ethanol^b^	etE015				
*Plectranthus hadiensis* (Forssk.) Schweinf. ex Sprenger	Diethyl ether^d^	dietE016	Lamiaceae	Kibwankulata	Leaves	AG210 (Makerere University Herbarium, Uganda)
	*n*-hexane^d^	hE016				

* Specimens have been digitized and are available for viewing at http://sernecportal.org/portal/;

a Collected in Apr. 2016

b Collected in Oct. 2015

c Collected in Sep. 2013

d Collected in Sep. 2016

sox., Soxhlet extraction; sox. succ., successive Soxhlet extraction.

### Heme Biocrystallization Assay Library Screen

A library screen for *in vitro* inhibition of hemozoin formation was conducted to identify promising extract candidates for the antiplasmodial follow-up studies. Here, a modified heme biocrystallization assay served as an alternative technique to circumvent initial testing of antimalarial activity in parasite cultures. The assay does not replace experiments with plasmodia but may serve as a prescreen followed by antiplasmodial experiments.

β-hematin, which is structurally and chemically identical to hemozoin, was included in the heme biocrystallization assay. The extract library was first screened at a concentration of 1,000 µg/ml. Extracts displaying a hemozoin formation percent inhibition above 20 were tested at the next lower concentration (100 µg/ml). Extracts showing no hemozoin formation inhibition activity or inhibition values less than 20% were eliminated from the antimalarial investigations. Next, extracts exhibiting inhibition values above 1% were regarded as active and subsequently tested at 10 µg/ml. This experimental step was repeated at a final concentration of 1 µg/ml. Results are shown in Table 2.

**TABLE 2:** Results of the heme biocrystallization library screen; +: observed inhibition activity; -: negative; nt indicates that a sample was not tested.

Scientific name	Extract ID	Inhibition of *in vitro* hemozoin formation														
		1,000 µg/ml		100 µg/ml			10 µg/ml					1 µg/ml				
		20–50 %I	>50 %I	1–20 %I	20–50 %I	>50 %I	1–20 %I	20–50 %I		>50 %I		1–20 %I	20–50 %I		>50 %I	
*Securidaca longipedunculata*	eE001	−	−													
	wE001	−	−													
	hE001	nt														
	mE001	−	−													
	smE001	−	−													
*Microgramma lycopodioides*	eE002	nt														
	wE002	−	−													
	smE002	−	−													
*Ficus saussureana*	eE003	−	−													
	wE003	−	−													
	hE003	−	−													
	mE003	−	−													
	smE003	−	−													
*Sesamum calycinum* subsp. *angustifolium*	eE004	+	−	+	−	−	+		−		−	−		−		−
	wE004	−	−													
	hE004	+	−	−	+	−	−		+		−	−		+		−
	mE004	−	−													
	smE004	+	−	−	−	−										
*Leucas calostachys*	eE005	+	−	+	−	−	−		+		−	+		−		−
	wE005	−	−													
	hE005	−	+	+	−	−	−		−		−					
	smE005	−	+	−	−	−										
*Solanum aculeastrum*	eE006	−	−													
	wE006	−	−													
	hE006	−	−													
	smE006	−	−													
*Albizia coriaria*	eE007	−	−													
	etE007	−	−													
*Erythrina abyssinica*	eE008	−	+	−	−	−										
	etE008	+	−	−	−	−										
*Zanthoxylum chalybeum*	eE009	+	−	+	−	−	+		−		−	−		−		−
	etE009	+	−	+	−	−	+		−		−	−		−		−
	etE017	−	−													
	dietE017	nt														
	etE017a	−	−													
	dietE017a	−	−													
*Toddalia asiatica*	eE010	nt														
	etE010	−	−													
	dietE010	−	−													
	etE010a	−	−													
*Harungana madagascariensis*	eE011	−	−													
	etE011	+	−	−	−	−										
	dietE011	−	−													
	etE011a	−	−													
*Morella kandtiana*	etE012	−	−													
	etE012a	−	−													
	dietE012	nt														
*Cassine buchananii*	eE013	nt														
	etE013	−	−													
	etE013a	−	−													
*Warburgia ugandensis*	etE014a	−	−													
	dietE014	+	−	−	−	+	−		+		−	−		−		−
*Combretum molle*	eE015	−	−													
	etE015	−	−													
*Plectranthus hadiensis*	dietE016	+	−	−	−	−										
	hE016	−	+	+	−	−	−		+		−	+		−		−
Chloroquine diphosphate	Positive control	−	+	−	−	+	−		−		+	−		−		+
DMSO	Solvent control	−	−													

The seven extracts from five plant species that inhibited heme biocrystallization at 10 µg/ml and below were selected for further pharmacological investigation and subsequently included in the *in vitro* antiplasmodial and cytotoxicity dose-response experiments. These seven extracts were the *n*-hexane and the ethyl acetate extracts of *S. calycinum* subsp. *angustifolium* leaves (hE004, eE004), the ethyl acetate extract of *L. calostachys* leaves (hE005), the ethanolic and ethyl acetate extracts of *Z. chalybeum* stem bark (etE009, eE009), the *n*-hexane extract of *P. hadiensis* leaves (hE016), and the diethyl ether extract of *W. ugandensis* stem bark (dietE014).

### *In vitro* Antiplasmodial Activity

Seven extracts from five species identified by the hemozoin formation inhibition library screening were further tested for antiplasmodial activity against the chloroquine-resistant *P. falciparum* K1 strain. The results are shown in Table 3. Generally, the results obtained from the heme biocrystallization assay were supported by the antiplasmodial experiments, since all seven extracts exhibited high to moderate antiplasmodial activity, reaching half maximal inhibitory concentrations (IC_50_ values) below 25 µg/ml.

**TABLE 3:** Results of the *in vitro* dose-response studies, investigating the antiplasmodial activity and cytotoxic effects of selected medicinal plant samples from the Greater Mpigi region against chloroquine-resistant *P. falciparum* K1 and human MRC-5_SV2_ lung fibroblasts.

Extract ID	Plant species	Type of extract	*P. falciparum* K1	MRC-5_SV2_ cells	SI
			IC_50_ ± SEM	CC_50_ ± SEM	
dietE014	*Warburgia ugandensis*	Diethyl ether	0.5 ± 0.1	0.3 ± 0.1	0.6
eE005	*Leucas calostachys*	Ethyl acetate	5.7 ± 1.2	14.7 ± 2.9	2.6
eE009	*Zanthoxylum chalybeum*	Ethyl acetate	11.9 ± 3.7	24.1 ± 1.3	2.0
etE009	*Zanthoxylum chalybeum*	Ethanol	12.4 ± 2.4	26.9 ± 1.0	2.2
hE004	*Sesamum calycinum* subsp. *angustifolium*	*n*-hexane	19.6 ± 5.0	26.6 ± 3.7	1.4
eE004	*Sesamum calycinum* subsp. *angustifolium*	Ethyl acetate	21.9 ± 6.8	27.0 ± 3.8	1.2
hE016	*Plectranthus hadiensis*	*n*-hexane	23.1 ± 3.9	7.3 ± 1.1	0.3
Chloroquine	Positive control	- (Pure compound)	0.04 ± 0.0	>20	>500
Tamoxifen	Positive control	- (Pure compound)	Nt	3.85 ± 0.14	-
DMSO	Solvent control	- (Pure compound)	>64	>64	-

The half maximal inhibitory concentration (IC_50_) and the half maximal cytotoxic concentration (CC_50_) are stated in µg/ml. SEM, standard error of the mean; SI, selectivity index.

With an IC_50_ value of 0.5 µg/ml, the extract dietE014 (diethyl ether extract of *W. ugandensis* stem bark) showed the highest antiplasmodial activity against the tested strain of malaria parasite. The ethyl acetate extract of *L. calostachys* leaves (eE005) also exhibited strong antiplasmodial effects with a calculated IC_50_ value of 5.7 µg/ml. The IC_50_ values of extracts eE009, etE009 hE004, eE004, and hE016 ranged from 11.9 to 23.1 µg/ml, whereas the *n*-hexane extract of *P. hadiensis* leaves displayed the lowest antimalarial activity among the plant extract candidates tested.

### Cytotoxicity Counterscreen and Selectivity Index

In an effort to assess the cytotoxicity of the five antimalarial plant species, the seven pharmacologically active extracts previously introduced to the antiplasmodial assay were further studied in a human lung fibroblast toxicity assay using the MRC-5_SV2_ cell line. The results of this counterscreen are shown in Table 3 along with the calculated selectivity indices (SIs) for antiplasmodial activity. The SI enables for characterization and optimization of efficacy and safety of drug candidates and is regularly used as a vital parameter in drug discovery for assessing whether a safety-efficacy profile is appropriately balanced for a given indication (Muller and Milton, 2012; Babii et al., 2018; Zhang et al., 2019; Touret et al., 2020).

The majority of extracts displayed moderate cytotoxic effects and half maximal cytotoxic concentrations (CC_50_ values) above 10 µg/ml, ranging from 27.0 µg/ml (eE004) to 14.7 µg/ml (eE005). However, the diethyl ether extract of *W. ugandensis* stem bark (dietE014), which was the most active extract in the antiplasmodial experiments, showed strong cytotoxic effects on the human lung cells, displaying a CC_50_ value as low as 0.3 µg/ml. The recorded antiplasmodial activity of these extracts was found not to be selective (SI < 10) which may relate to the compositional complexity of the extracts.

### Investigation of the Mutagenic Effects of Extracts

The plant extract library was further screened for genotoxicity and mutagenic effects *via* the *Salmonella* reverse mutation assay without metabolic activation to detect direct mutagenic extracts at 500 µg/plate. The strains used were the *S. enterica* subsp. *enterica Typhimurium* strains TA98 and TA100. Strain TA98 is particularly susceptible to frameshift mutations, whereas strain TA100 is optimized for base-pair substitution mutations (Isono and Yourno, 1974; Jurado et al., 1993; Mortelmans and Zeiger, 2000). The results of the library screen for mutagenic effects of extracts (without metabolic activation) are given in Table 4, showing the calculated mutagenicity indices (MIs) and the MI interpretations. Absolute data such as mean values of His+ revertant colonies are provided in Supplementary Table S1.

**TABLE 4:** Results of the *Salmonella* reverse mutation assay showing the mutagenicity indices (MIs) and the MI interpretations at 500 µg/plate; +: positive, mutagenic; +/−: weakly mutagenic; −: negative: non-mutagenic; nt: not tested; GI: growth inhibition.

Scientific name	Extract ID	TA98				TA100			
		Without metabolic activation		With metabolic activation		Without metabolic activation		With metabolic activation	
		Mutagenicity	MI	Mutagenicity	MI	Mutagenicity	MI	Mutagenicity	MI
*Securidaca longipedunculata*	eE001	**−**	0.8	**−**	0.9	**−**	1.4	**−**	0.9
	wE001	**−**	1.5	**−**	0.7	**−**	1.6	**−**	1.2
	hE001		nt	**−**	0.4		nt	**−**	0.8
	mE001	**−**	0.7	**−**	1.1	**−**	1.2	**−**	0.4
	smE001	**−**	0.9	**−**	1.0	**−**	1.5	**−**	0.0
*Microgramma lycopodioides*	eE002	**−**	1.2	**−**	0.9	**−**	1.4	**−**	1.0
	wE002	**−**	1.2	**−**	1.1	**−**	1.1	**−**	0.7
	smE002	**−**	1.4	**−**	0.8	**+/−**	1.7		1.0
*Ficus saussureana*	eE003	**−**	0.9	**−**	0.9	**−**	1.0	**−**	0.7
	wE003	**−**	1.0	**−**	0.8	**−**	1.6	**−**	1.0
	hE003	**−**	1.1	**−**	0.7	**−**	1.5	**−**	0.9
	mE003	**−**	0.8	**−**	0.8	**−**	1.4	**−**	1.0
	smE003	**−**	1.0	**−**	0.7	**−**	1.6	**−**	0.9
*Sesamum calycinum* subsp. *angustifolium*	eE004	**−**	0.8	**−**	0.8	**−**	1.3	**−**	1.0
	wE004	**−**	1.0	**−**	1.0	**+/−**	1.7	**−**	1.1
	hE004	**+**	6.5	**−**	0.6	**−**	1.5	**−**	1.0
	mE004	**−**	1.0	**−**	1.1	**−**	1.4	**−**	1.0
	smE004	**−**	0.7	**−**	1.0	**−**	1.5	**−**	1.1
*Leucas calostachys*	eE005	**−**	0.9	**−**	0.6	**−**	1.4	**−**	0.7
	wE005	**−**	1.0	**−**	0.9	**+/−**	1.7	**−**	1.1
	hE005	**−**	1.2	**−**	0.8	**−**	1.5	**−**	1.0
	smE005	**−**	1.0	**−**	0.8	**−**	1.6	**−**	1.0
*Solanum aculeastrum*	eE006	**−**	0.8	**−**	0.8	**+/−**	1.9	**−**	1.0
	wE006		nt	**−**	1.1		nt	**−**	1.0
	hE006	**+**	10.7	**−**	0.6	**−**	1.6	**−**	0.9
	smE006	**−**	0.8	**−**	0.7	**+/−**	1.7	**−**	1.0
*Albizia coriaria*	eE007	**−**	0.7	**−**	0.9	**−**	1.3	**−**	1.0
	etE007	**−**	1.1	**−**	1.0	**−**	1.6	**−**	1.0
*Erythrina abyssinica*	eE008	**−**	0.8	**−**	0.4	**−**	1.0	**−**	0.8
	etE008	**−**	1.0	**−**	0.4	**−**	1.0	**−**	0.7
*Zanthoxylum chalybeum*	eE009	**−**	1.1	**−**	1.0	**+/−**	1.8	**−**	1.0
	etE009	**−**	0.8	**−**	1.0	**−**	1.5	**−**	1.0
	etE017		nt		nt		nt		nt
	dietE017	**−**	0.7		nt	**+/−**	1.8		nt
	etE017a	**−**	0.9	**−**	0.9	**−**	1.4	**−**	1.4
	dietE017a	**−**	0.7	**−**	0.7	**−**	1.2	**−**	0.9
*Toddalia asiatica*	eE010	**+**	3.8	**−**	0.9	**+**	3.9	**−**	1.0
	etE010	**+/−**	1.8	**−**	0.4	**+**	3.1	**−**	0.7
	dietE010	**+**	4.0	**−**	0.6	**+**	3.7	**−**	1.2
	etE010a	**+/−**	1.7	**−**	1.0	**+**	3.2	**−**	0.9
*Harungana madagascariensis*	eE011	**−**	1.0	**−**	0.9	**−**	1.4	**−**	1.0
	etE011	**−**	0.9	**−**	0.6	**−**	1.4	**−**	1.2
	dietE011	**−**	0.7	**−**	0.4	**−**	1.1	**−**	0.6
	etE011a	**−**	1.3	**−**	0.8	**−**	1.4	**−**	0.7
*Morella kandtiana*	etE012	**−**	0.8	**−**	1.1	**−**	1.5	**−**	1.0
	etE012a	**−**	1.0	**−**	1.1	**−**	1.4	**−**	0.9
	dietE012	**−**	1.0	**−**	0.8	**−**	0.7	**−**	0.9
*Cassine buchananii*	eE013	**−**	0.9	**−**	0.8	**−**	1.6	**−**	1.1
	etE013	**−**	0.9	**−**	1.1	**−**	1.5	**+/−**	1.7
	etE013a	**−**	0.9	**−**	0.9	**−**	1.3	**−**	0.9
*Warburgia ugandensis*	etE014a	**−**	0.8	**−**	0.9	**−**	0.7	**−**	1.3
	dietE014		nt		nt		nt		nt
*Combretum molle*	eE015	**−**	0.9	**−**	1.1	**−**	1.1	**−**	1.2
	etE015	**−**	1.0	**−**	1.2	**−**	1.5	**−**	1.0
*Plectranthus hadiensis*	dietE016	**−**	1.0	**−**	1.1		nt	**−**	1.2
	hE016	**−**	0.9	**−**	0.5	**−**	1.4	**−**	0.8
Spontaneous mutations	Negative control	**−**	1.0	**−**	1.0	**−**	1.0	**−**	1.0
2-Nitrofluoren	Positive control	**+**	18.9		nt		nt		nt
Methyl methanesulfonate	Positive control		nt		nt		6.6		nt
2-Aminoflourene	Positive control	**−**	1.0	**+**	12.4	**+**	2.2	**+**	6.1

Among the 56 extracts from 16 plant species, four extracts from three species were identified to cause significant direct mutagenic effects in the TA98 strain at a concentration of 500 µg/plate with MIs ranging from 3.8 to 10.7. These extracts were the *n*-hexane extract of *S. calycinum* subsp. *angustifolium leaves* (hE004, MI: 6.5; previously active in the antiplasmodial experiments against *P. falciparum* K1), the *n*-hexane extract of *Solanum aculeastrum* roots (hE006, MI: 10.7), and the diethyl ether and ethyl acetate extracts of *Toddalia asiatica* leaves and bark (eE010, MI: 3.6; dietE010; MI: 4.0). Two more extracts of *T. asiatica* showed weak mutagenicity against the TA98 strain (without metabolic activation). These were the ethanolic extracts etE10 (MI: 1.8) and etE010a (MI: 1.7), resulting in all four *T. asiatica* extracts displaying either significant or weak genotoxicity against the TA98 strain.

Interestingly, these four extracts of *T. asiatica* also exhibited significant mutagenic effects during the experiments with the TA100 strain at 500 µg/plate, and mutation rates were tripled and almost quadrupled. The MIs ranged from 3.1 to 3.9 (MI_eE010_: 3.9; MI_etE010_: 3.1; MI_dietE010_: 3.7; MI_etE010a_: 3.2). No other extracts were identified as causing significant mutagenicity in the TA100 strain. However, seven extracts from five plant species displayed weak mutagenic effects. These were, in descending order of the MI: 1) extract eE006 (ethyl acetate extract of *S. calycinum* subsp. *angustifolium* leaves, MI: 1.9); 2) and 3) extracts eE009 and dietE017 (ethyl acetate and diethyl ether extracts of *Z. chalybeum* stem bark, MIs: 1.8); 4)–7) extracts smE002 (methanolic extract of *Microgramma lycopodioides* rhizomes, MI: 1.7), wE004 (aqueous extract of *S. calycinum* subsp. *angustifolium* leaves, MI: 1.7), wE005 (aqueous extract of *L. calostachys* leaves, MI: 1.7), and smE006 (methanolic extract of *S. aculeastrum* roots, MI: 1.7).

For further illustration, plate photos of revertant colonies for extract eE007 (*Albizia coriaria*, non-mutagenic against test strain TA98) and hE006 (*S. aculeastrum*, mutagenic against test strain TA98) are provided in Supplementary Figure S1.

### Simulation of Human Liver Metabolism and Mutagenic Effects

The experiments for investigation of genotoxicity of the 56 extracts were repeated after incorporating a pre-incubation assay, aiming at the *in vitro* simulation of human liver metabolism and the assessment of a potential bioactivation or deactivation/detoxification of mutagenic compounds in the extracts. Prior to the *Salmonella* reverse mutation assay, the extracts were treated with a pooled hepatic S9 fraction, which is the post-mitochondrial supernatant fraction of homogenized liver. It was prepared by homogenization of human liver in isotonic KCl with subsequent separation by centrifugation at 9,000 g (Hamel et al., 2016). The human liver S9 fraction represents a rich source of drug metabolizing phase I and phase II enzymes, including the cytochromes P450, UDP-glucuronosyltransferases, acetyltransferases, methyltransferases, glutathione S-transferases, sulfotransferases, epoxide hydrolases, carboxylesterases, and flavin-monooxygenases. It is therefore widely used in the study of drug interactions and xenobiotic metabolism (Jia and Liu, 2007; Cox et al., 2016; Vrbanac and Slauter, 2017).

Results, including calculated MIs, are given in Table 4. Mean values of His+ revertant colonies are reported in Supplementary Table S1. None of the extracts previously identified in the screening without metabolic activation as mutagenic retained their genotoxic activity after pre-incubation of the plant extract library with human S9 liver fraction at a concentration of 500 µg/plate. This observation was irrespective of the strain tested and whether extracts displayed significant or weak mutagenic properties in the experiments without metabolic activation. In addition, none of the other extracts in the library displayed significant mutagenicity in both strains after metabolic activation. Only one extract, the ethanolic extract of *Cassine buchananii* stem bark (etE013), exhibited a weak genotoxic effect on test strain TA100 after contact and treatment with the human hepatic S9 fraction, reporting an MI of 1.7.

## Discussion

In this study, seven extracts from five plant species used in the Ugandan Greater Mpigi region in the treatment of malaria were identified from a library of 56 extracts and selected for antiplasmodial follow-up investigations due to their hemozoin formation inhibition activity in the *in vitro* heme biocrystallization assay. The extracts that were further studied were extracts hE004 and eE004 (an *n*-hexane and an ethyl acetate extract of *S. calycinum* subsp. *angustifolium* leaves), eE005 (an ethyl acetate extract of *L. calostachys* leaves), etE009 and eE009 (an ethanolic and an ethyl acetate extract of *Z. chalybeum* stem bark), hE016 (an *n*-hexane extract of *P. hadiensis* leaves), and dietE014 (a diethyl ether extract of *W. ugandensis* stem bark). The modified heme biocrystallization assay proved to be an effective method for pre-screening of natural product libraries since all seven extracts subsequently displayed significant antimalarial activity in the antiplasmodial experiments against chloroquine-resistant *P. falciparum* K1. The results of this study therefore further add to the scientific basis for their effectiveness in traditional use in the Greater Mpigi region in Uganda as previously described (Schultz et al., 2020b). Furthermore, antimalarial activity was studied and verified for the first time for the majority of the species investigated, which was previously determined by the DoP method (Schultz et al., 2021a). It needs to be pointed out that only those extracts were selected for antiplasmodial follow-up experiments that acted as hemozoin formation inhibitors in the pre-screen at concentrations of 10 µg/ml or lower. It is therefore possible that extracts of other species in the library might also possess antimalarial properties on basis of other mechanisms of action, which were not covered in this study.

The strongest antiplasmodial activity was displayed by the diethyl ether extract of *W. ugandensis* stem bark (dietE014), showing an IC_50_ value as low as 0.5 µg/ml. The plant is an evergreen tree, also known as the pepper-bark tree and East African greenheart, that grows in drier highland forest and lower rainforest areas throughout East Africa (Katende et al., 1995; Dharani and Yenesew, 2010; Dharani, 2019). In the Sango bay area in Southern Uganda, it is considered a threatened species by the locals due to poor harvesting techniques and unsustainable harvesting intensities of the stem bark (Ssegawa and Kasenene, 2007). However, in addition to the strong antimalarial properties displayed by extract dietE014, the results of the cytotoxicity experiments against MRC-5_SV2_ lung fibroblasts indicate even more potent cytotoxicity (CC_50_: 0.3 µg/ml). This led to a relatively low calculated SI of 0.6, making this plant species and its extract potentially less suitable for selection for further studies on the isolation and discovery of novel antimalarial drug leads. In another study, the leaves of *W. ugandensis* were reported to exhibit comparable cytotoxic activity against brine shrimp larvae (LC_50_: 24.5 µg/ml) as cyclophosphamide (LC_50_: 16.3 µg/ml), a standard anticancer drug that was used as a positive control (Mbwambo et al., 2009). *W. ugandensis* was also cited as being used by Ugandan healers in traditional therapy of several types of cancer (breast cancer, cervical cancer, intestinal cancer, prostate cancer, skin cancer, throat cancer, and leukemia) in the Greater Mpigi region (Schultz et al., 2020b). Therefore, it will be interesting to further investigate the plant’s cytotoxic properties in selectively destroying related cancer cells. Interestingly, extract dietE014 could not be evaluated for genotoxicity because it displayed growth inhibitory activity against both *Salmonella* strains used in this study. These anti-*Salmonella* properties have previously been described for apolar extracts of *W. ugandensis* stem bark from Kenya with MIC values ranging from 31 µg/ml to 488 µg/ml, depending on the *Salmonella* strain (Peter et al., 2015). In another study, antiplasmodial properties were reported for chloroform, ethyl acetate, aqueous, and methanolic extracts of *W. ugandensis* stem bark against *P. knowlesi* (Were et al., 2010). The most promising extract in this study was the apolar chloroform extract with an IC_50_ value of 3.1 µg/ml. In the same study, this extract was further investigated for chemosuppression of *P. berghei* growth in BALB/c mice at 200 mg/kg/day, reaching *in vivo* chemosuppression of 69% (curative) and 49% respectively (prophylactic). However, all mice treated with the chloroform extract had died by the end of the assays (12 days) when used for prophylaxis, whereas no deaths were observed in uninfected mice and the positive controls. Another study investigating *W. ugandensis* stem bark, harvested in Ethiopia, tested extracts (petroleum ether, dichloromethane, acetone, methanolic) against chloroquine-sensitive *P. falciparum* 3D7 (Wube et al., 2010). The petroleum ether and dichloromethane extracts exhibited strong antiplasmodial activity (IC_50_: 6.9 and 8.1 µg/ml) whereas the acetone and methanolic extracts were inactive. Both pharmacologically active extracts showed cytotoxic effects on KB cells (ED_50_: 2.7 and 5.6 µg/ml), thus achieving a less promising SI. Six coloratane and six drimane sesquiterpenes were further isolated from the dichloromethane extract, of which two compounds exhibited some plasmodicidal activity against the chloroquine-resistant *P. falciparum* K1 strain (IC_50_: 7.3 and 7.9 µM). None of the isolated compounds tested were assessed for cytotoxicity in the study. In the literature, there are also a few studies investigating the antileishmanial activity of *W. ugandensis*, which were initiated due to its widespread traditional use to treat this neglected disease in Kenya (Ngure et al., 2009a; Ngure et al., 2009b; Githinji et al., 2010).

The extract exhibiting the second strongest antiplasmodial activity against chloroquine-resistant *P. falciparum* K1 in the plant extract library was the ethyl acetate extract eE005, obtained from *L. calostachys* leaves, achieving an IC_50_ value of 5.7 µg/ml. Sample eE005 was also the most promising extract in the study due to its relatively low cytotoxicity against MRC-5_SV2_ cells (CC_50_: 14.7 µg/ml). Consequently, a SI of 2.6 was calculated which was the highest selectivity index in this study. Interestingly, the same plant extract eE005 was recently identified by the authors as a strong selective cyclooxygenase-2 (COX-2) inhibitor (IC_50_: 0.66 μg/ml) with a promising selectivity ratio (COX-2/COX-1) of 0.1 (Schultz et al., 2021c). Potentially generating fewer side effects due to decreased COX-1 and increased COX-2 inhibition, sample eE005 seemed to be much more potent in the study than comparable commercial COX-2 inhibitor drugs, such as Aspirin and ibuprofen. At the same time, eE005 only displayed low inhibitory activity against *S. aureus* (MIC: 500 μg/ml) and no antibacterial growth inhibition effects on multidrug-resistant *Listeria innocua*, *Escherichia coli* (MICs: > 500 μg/ml), *Enterococcus faecium*, *Klebsiella pneumoniae*, *Acinetobacter baumannii*, *Pseudomonas aeruginosa*, and *Enterobacter cloacae* (MICs: > 256 μg/ml) at the highest concentrations tested (Schultz et al., 2020a; Schultz et al., 2021c). Moreover, the *n*-hexane extract of the leaves (hE005) showed significant quorum quenching activity against the accessory gene regulator (*agr*) system in *S. aureus* (Schultz et al., 2020a). *L. calostachys* was recently identified using the DoP method as a highly understudied medicinal species that merits further ethnopharmacological and pharmacognostic research in the lab (Schultz et al., 2021a). In fact, only three other studies have been published that examine potential bioactive properties of the plant. All of these focus on the potential antiplasmodial properties of *L. calostachys* crude extracts. The first bioactivity study reported no significant antiplasmodial activity of *L. calostachys* (Muregi et al., 2004) while the second study reported antiplasmodial activity of a methanolic extract (IC_50_: 3.45 μg/ml) and an aqueous extract (IC_50_: 0.79 μg/ml) against *P. knowlesi* (Nyambati et al., 2013). The last of these studies reported low antiplasmodial activity of a chloroform and a methanolic extract against chloroquine-sensitive *P. falciparum* D6 (IC_50_: 40.2 and 88.4 μg/ml) (Jeruto et al., 2015). The species *L. calostachys* was recently reviewed more in detail in the discussion sections of two of our recent publications (Schultz et al., 2021a; Schultz et al., 2021c). As far as to the author’s knowledge, there have been no studies published on the identification or isolation of pharmacologically active secondary plant metabolites from *L. calostachys* to date, highlighting the vital need to further study this interesting medicinal plant.

According to the ethnobotanical survey among Ugandan traditional healers (Schultz et al., 2020b), the traditional methods of preparation most often cited were boiling ground plant parts in water (aqueous decoctions) or suspending in water (cold infusions), followed by oral administration. Aqueous extracts were therefore included into this study. However, other types of solvents were included in a “pre-fractionation” process during initial extraction procedures in order to investigate not only the chemistry yielded by traditional preparation but also chemistries only accessible by alternative extraction methods. The aim of this strategy was to depict the full range of polarity during the extractions (from aqueous extracts *via* methanolic, ethanolic, ethyl acetate, diethyl ether to *n*-hexane extracts with decreasing polarity), using new, unextracted material for each type of extraction. Thus, plant material and individual extracts derived from it were regarded as chemical libraries that merit pharmacological investigation of the totality of ingredients provided. Similar to the results of pharmacological studies of extracts derived from the library that were published previously (Schultz et al., 2020a; Schultz et al., 2021c), the apolar extracts exhibited the strongest antimalarial effects in the *in vitro* assays. This phenomenon might be explained by the fact that the extracts produced were fine-filtered prior to evaporation of the solvent and drying of extracts, ultimately leading to the removal of tiny solids that would still be present in traditional preparations. Aqueous decoctions and infusions with ground plant material are usually not filtered by the traditional healers, meaning that patients ingest these tiny solids. It is likely that apolar antimalarial secondary plant metabolites remain in the traditional herbal remedy as a result; hence, these active ingredients only occur in the apolar extracts screened in this study. Another possible explanation could be that lipophilic compounds are also extracted to a certain degree during boiling (potentially assisted by bipolar surface-active secondary metabolites present in the plant material).

In a previous screening of the same plant extract library for antiinflammatory activity, nine extracts were identified as potent COX-2 inhibitors (Schultz et al., 2021c). Interestingly, five of the seven extracts that were reported as having strong antiplasmodial activity in the present study were among the nine COX-2 inhibiting extracts (dietE014, eE005, etE009, hE004, eE004). In addition, other active antimalarial extracts were eE009 and hE016, derived from *Z. chalybeum* and *P. hadiensis*, whereas etE009 and dietE016 acted as COX-2 inhibitors. Thus, all five medicinal plant species reported to possess antiplasmodial activity also showed significant inhibition of COX-2. This suggests that the potential molecular mechanism of action may be similar. Heme plays a major role in both assays. On the one hand, heme is a vital co-factor for COX isoenzymes and has been introduced externally to the COX reaction in the antiinflammatory assays (Chandrasekharan and Simmons, 2004; Schultz et al., 2021c). On the other hand, toxic free heme is released during plasmodial degradation of hemoglobin and subsequently detoxified by heme biocrystallization (Schmitt et al., 1993; Roy, 2017). Due to the heme biocrystallization pre-screen conducted in this study, it is likely that inhibition of hemozoin formation is the mechanism of action for the reported antiplasmodial activity of plant extracts. In past studies on the antimalarial drug chloroquine, scientists hypothesized that the hemozoin formation is inhibited due to the drug’s ability to establish complexes with free heme (Cohen et al., 1964; Chou et al., 1980; Egan, 2001; Egan, 2004). This has been confirmed in a recent study by (Kapishnikov et al., 2019), in which the mode of action of quinoline antimalarial drugs in red blood cells infected by *P. falciparum* was revealed *in vivo* using correlative X-ray microscopy. The authors report that an excess of drug molecules in the digestive vacuole of the parasite leads to formation of a complex with the free heme, making it unavailable for biocrystallization. The drug molecule also covers and blocks a substantial number of the available docking sites on the surface of the hemozoin crystals that are formed in the digestive vacuole of the parasite. These processes cause membrane puncture and spillage of heme into the interior of the parasite due to the complex being driven toward the digestive vacuole membrane (Kapishnikov et al., 2019). In terms of the COX-2 inhibition activity of the medicinal plant extracts from the Greater Mpigi region, potential chelation of the co-factor free heme and complex formation with active secondary metabolites may be responsible for the reported inhibition of COX-2 and resultant antiinflammatory properties.

Genotoxicity is a major cause of the initiation and development of many types of cancer. It is defined as the ability of different substances to produce damage to genetic material, i.e., to cause DNA mutations but also to damage those cellular components that are responsible for the functionality and behavior of chromosomes within the cell (Bhattachar, 2011; Nagarathna et al., 2013; Słoczyńska et al., 2014). Therefore, genotoxicity assays are *in vitro* and *in vivo* tests that have been developed to detect genotoxic, mutagenic, and potentially carcinogenic substances that induce genetic damage, point mutations in genes, large deletions or rearrangements of DNA, cellular transformation, and chromosomal breakage (Tice et al., 2000; Tejs, 2008; Samiei et al., 2015). Unlike marketed drug compounds in the pharmaceutical industry, plants used in traditional medicine systems have often never been investigated for potential genotoxic effects (Verschaeve, 2015). To increase awareness of potential health hazards and to determine the safety of traditionally used herbal remedies, the plant extracts were studied using the widely accepted *Salmonella* reverse mutation assay (Ames test). Plants with mutagenic properties should be considered potentially unsafe, especially for long-term use (Verschaeve, 2015). In the European Union, the Ames test is used as part of well described strategies as an initial experimental method for assessment of the short-term genotoxicity and mutagenicity of chemical agents. With identification of potential mutagens (“positive” results) using the Ames test, further studies with different *in vitro* and *in vivo* assays are conducted for a detailed understanding and confirmation of carcinogenic effects (Samiei et al., 2015; Verschaeve, 2015). The Ames test employs several histidine dependent bacterial strains of *Salmonella* to identify agents that are capable of causing genetic damage that leads to gene mutations. These strains possess preexisting mutations in the histidine operon, acting as hot spots for mutagens, causing DNA damage *via* different mechanisms of action (Ames et al., 1973; Mortelmans and Zeiger, 2000). When grown on minimal media agar plates in the presence of mutagens, the genes’ function for cells synthesizing histidine may be restored (reverse mutation) and the mutated cells form colonies that are counted (Mortelmans and Zeiger, 2000; Tejs, 2008).

The experiments for assessment of potential procarcinogenic properties of plant extracts *via* evaluation of *in vitro* mutagenicity and genotoxicity identified four extracts from three species that caused significant direct mutagenic effects against test strain TA98 and four extracts from one species with significant direct mutagenic effects against test strain TA100. The species *T. asiatica* showed the most significant genotoxic effects on both test strains (without metabolic bioactivation at a concentration of 500 µg/plate). TA98 is susceptible to frameshift mutations and the diethyl ether and ethyl acetate extracts of *T. asiatica* leaves and bark (eE010, MI: 3.6; dietE010, MI: 4.0) resulted in high genotoxicity. Two additional extracts of this species also displayed weak mutagenicity (etE10, MI: 1.8; etE010a, MI: 1.7). In addition, all four extracts of *T. asiatica* exhibited strong mutagenic effects on the TA100 test strain at 500 µg/plate, nearly quadrupling the (base-pair substitution) mutation rates. The woody liana or shrub *T. asiatica* is a commonly used medicinal plant throughout Africa and Southeast Asia. It has previously been identified as a “highly studied” medicinal plant species using the DoP method (Dharani and Yenesew, 2010; Schultz et al., 2021a). As far as the authors are aware, no study has yet assessed the potential genotoxic effects of *T. asiatica*, making this study the first report of mutagenic effects for the species. The fact that it is widely used in different traditional medicine systems, and that, after *S. longipedunculata,* it is the second most studied species in the plant extract library justifies further future investigations assessing its toxicity and potential carcinogenic properties using more advanced toxicological methods. Plant extracts are complex mixtures, and it is difficult to speculate which of the secondary plant metabolites are responsible for the mutagenic effects of some of the extracts. The results of this study warrant the phytochemical characterization of potentially genotoxic plant extracts and the isolation of mutagenic compounds.

Without metabolic bioactivation, the *n*-hexane extract of *S. calycinum* subsp. *angustifolium* leaves (hE004) produced the strongest genotoxic effects measured in this study, reaching a calculated MI of 6.5 when being in contact with the TA98 test strain. The absence of mutagenic activity against test strain TA100 and after metabolic bioactivation against both strains indicates that frameshift mutations were caused and that genotoxic secondary plant metabolites were successfully detoxified/deactivated by human liver enzymes. Interestingly, extract hE004 was also the fifth most active extract in the evaluation of the library for antiplasmodial activity against *P. falciparum* K1 (IC_50_ value: 19.6 μg/ml; TI: 1.4). The antimalarial activity of *S. calycinum* subsp. *angustifolium* leaves was reported for the first time in this study, which represents another scientific evidence supporting its therapeutic use in the Ugandan Greater Mpigi region. The authors previously stated that extract hE004 acts as a potent antiinflammatory COX-2 inhibitor *in vitro* (IC_50_ value: 3.65 μg/ml) and as an *agr* system quorum sensing inhibitor [IC_50_ values: 2, 2, 16, and 32 μg/ml (*agr* I–IV)]. In these recent publications, *S. calycinum*subsp. *angustifolium* was extensively reviewed in the discussions (Schultz et al., 2020a; Schultz et al., 2021a; Schultz et al., 2021c).

Apart from extract hE004, none of the other extracts that were previously identified as possessing antimalarial effects showed significant mutagenic activity in the genotoxicity assays (with and without metabolic bioactivation). Interestingly, none of the mutagenic extracts from the screen without metabolic bioactivation retained their genotoxic activity after metabolic bioactivation of the plant extract library with human S9 liver fraction. What should be emphasized is that none of the 56 extracts in the library displayed significant genotoxic activity against either strain after metabolic bioactivation, indicating that effective deactivation of potential procarcinogens occurred during the *in vitro* simulation of human liver metabolism. Nevertheless, more thorough screening for potential harmful genotoxic effects *via* other methods is needed in order to recommend the plants used in the Greater Mpigi region as being safe for long-term use.

The ritual plant *P. hadiensis* was also investigated for genotoxicity due to the elevated prevalence of rapidly growing large breast masses in young women in the Nakawuka village area, Wakiso district, Uganda. As far as the authors are aware, this study is the first investigation of potential genotoxic properties of this plant. The results show that extracts of *P. hadiensis* had no mutagenic/genotoxic effects on either test strain regardless of whether extracts underwent metabolic bioactivation with human S9 liver fraction prior to assaying. On the contrary, previous studies have shown cytotoxic and anticancer effects of *P. hadiensis* (Minker et al., 2007; Mothana et al., 2010; Menon and Gopalakrishnan, 2015). For example, terpenoids isolated from the shoots induced apoptosis in human colon cancer cells *via* the mitochondria-dependent pathway (Menon and Gopalakrishnan, 2015). In the Greater Mpigi region, *P. hadiensis* has mainly been reported as being used to treat skin cancer (relative frequency of citation: 36%) as well as, with low informant consensus, abdominal cancer, leukemia, brain cancer, breast cancer, intestinal cancer, lung cancer, prostate cancer, throat cancer, and uterine cancer (Schultz et al., 2020b). The increased incidence of rapidly growing breast mass diagnoses (not necessarily cancer, but possibly) is 1) most likely associated with genetic predisposition; 2) could be the result of phytoestrogen properties of this plant; or 3) could be caused by some other unknown environmental stimulus. In the future, medical studies should emphasize the fact that inherited genetic variations are a common phenomenon in relatively isolated ethnicities and minority groups (Chlebowski et al., 2005; Brennan, 2017). However, phytoestrogens derived from *P. hadiensis* may contribute to more rapid growth of tumors, particularly benign masses. If *P. hadiensis* would be the cause alone, it would be very potent and most likely act genotoxic in the *Salmonella* reverse mutation screen. Yet, cancer development and cause are highly complex, and *P. hadiensis* could still act as an external stimulus in relatively isolated communities with high prevalence of inactivated tumor suppressor genes in a so-far unknown mechanism. Further research is needed to reliably rule out *P. hadiensis* as the causative agent for the rapidly enlarging masses observed in young female patients. In addition, it is important to recognize that potentially benign masses can grow rapidly and may mimic cancer from the health provider and patient’s perspective (Heilmann et al., 2012). This can result in erroneous treatment (e.g., mastectomy) in resource-limited settings unless tissue diagnosis is performed first.

Genotoxicity studies in general and the *Salmonella* reverse mutation assay in particular have some limitations that need to be discussed. First, some plant extracts may potentiate the genotoxicity of a known mutagen, making them become co-mutagenic. This also means that a plant extract may also have an antimutagenic action when administered with a known mutagen. Moreover, many carcinogens may possess genotoxic properties in one tissue but have antigenotoxic/anticarcinogenic properties in a different tissue, making investigations and experimental design complicated and complex (Verschaeve, 2015). The same applies to the dose of the drug (“the dose makes the poison”). Due to the high sensitivity of the *Salmonella* reverse mutation assay, the risk of false negatives is relatively low. However, the relatively low specificity means that false (misleading) positives may occur more often, which is problematic. Thus, further studies utilizing other *in vitro* methods, such as the mouse lymphoma L5178Y cell Tk (thymidine kinase) gene mutation assay (MLA), the micronucleus assay or the mammalian cell metaphase chromosome aberration assay, and *in vivo* follow-up, e.g., *via* the transgenic mouse test or the UDS test, should be conducted as recommended in the ICH guideline S2 (R1) issued by the European Medicines Agency (EMA, 2013; Verschaeve, 2015).

The present study can be regarded as a contribution to drug discovery since most of the past discoveries of drug leads taking the ethnopharmacological approach were based on *in vitro* screening studies from initial studies, which were then followed up on *via* bioassay-guided fractionation strategies, investigations of the mechanisms of action, and *in vivo* antimalarial and toxicity experiments. The study identified several medicinal plant species and extracts derived from them that displayed significant antiplasmodial *in vitro* activity. The obtained pharmacological data did provide early stage support for the therapeutic use of the medicinal plants in rural Uganda as all these plants are frequently used by traditional healers to treat malaria and related fever (Schultz et al., 2020b). The promising results obtained in this study suggest that such studies will be vital for future research work on the medicinal plants from the Greater Mpigi region. Yet, there may be synergistic effects between multiple active secondary plant metabolites which could potentially jeopardize bioassay-guided isolation efforts. After this initial screening study, further research work is also required to phytochemically characterize the bioactive extracts of the most promising antimalarial medicinal plant species.

All results of this study were reported back to the traditional healers who originally participated in the ethnobotanical survey, and a video article describing a two-day workshop method for transfer of lab results is currently in review with a scientific video journal (Schultz et al., 2021b). The authors believe that it is the responsibility of the ethnopharmacologist to facilitate bidirectional knowledge transfer and feedback once a study is completed.

## Materials and Methods

### Ethnobotanical Information

Ethnobotanical information, e.g., use reports, on the 16 medicinal plant species for medical treatment was obtained by means of a survey among 39 traditional healers from 29 different villages in the Greater Mpigi region in Uganda. The results of this study were previously published (Schultz et al., 2020b) and serve as a basis for the pharmacological experiments. Additional information on the ritual use of *P. hadiensis* in Nakawuka village (Wakiso District) has been obtained through anecdotal reports by local healthcare providers and female patients in 2016.

### Collection of Plant Material

Plant sample and voucher specimen material were collected under the guidance of the traditional healers in 2013, 2015, and 2016. Best practice collection procedures were followed at all times (Alexiades and Sheldon, 1996; Martin, 2004; Heinrich et al., 2018). Assignments of plant family correspond to The Angiosperm Phylogeny Group IV guidance (The Angiosperm Phylogeny, 2016). Plant identification and assignment of scientific names were performed following the current standards in the field of ethnopharmacology (Weckerle et al., 2018). Scientific names were cross-checked and taxonomically validated with https://www.theplantlist.org. Voucher specimens were prepared for all species investigated in this study and were deposited in indexed herbaria at the Makerere University Herbarium (MUH) in Kampala, Uganda. Select specimens were additionally deposited at the Emory University Herbarium (GEO) in Atlanta, GA, United States of America. These herbarium voucher specimens were made digitally available on the SERNEC portal (http://sernecportal.org/portal/index.php). Voucher specimen numbers are provided in Table 1.

### Preparation of Extracts and Extract IDs

The collected plant material was roughly chopped, then shade dried, and taken to the laboratory. Samples were ground prior to extraction. The extraction procedure has previously been described by Schultz et al. (2020a) (flow sheet in the Supplementary Figure S2 of the cited article). Briefly, the extraction methods applied were either maceration, Soxhlet extraction, or aqueous decoction. Extractions were performed using different solvents (water, methanol, ethanol, ethyl acetate, diethyl ether, *n*-hexane), enabling selective extraction of biomolecules of different polarities from the samples (“pre-fractionation” approach). New dried material was always used, except for the methanolic Soxhlet extractions which were performed successively from extracted dry material after Soxhlet extraction with *n*-hexane. This way, pharmacological investigation of the totality of ingredients provided by the individual medicinal plant was assured. All extracts were dried using a rotary vacuum evaporator or under a nitrogen stream and then stored at −20°C in the dark. Labeling of the crude extracts was performed according to their extraction solvent and the collection number (EXXX) that was assigned to each individual plant species during the field studies, ranging from E001 to E017. In terms of the maceration procedure, the extractants used were a) methanol (sample ID: mEXXX); b) ethanol (etEXXX); c) ethyl acetate (eEXXX); and d) diethyl ether (dietEXXX). Crude extracts produced *via* Soxhlet extraction were prepared using e) *n*-hexane (hEXXX); and f) methanol (smEXXX). In the Ugandan Greater Mpigi region, traditional healers often prepare and administer herbal drugs as aqueous decoctions (Schultz et al., 2020b). To simulate this original method of preparation, the plant material was additionally boiled in g) water at 95°C for 30 min while being stirred (wEXXX).

### Sample Preparation

Dry extracts were dissolved in DMSO (Carl Roth) at a sample concentration of 10 mg/ml, using a vortex mixer or a sonicator. For some samples, moderate or low solubility was observed at RT. These samples were further treated with sonication as well as a slow gradual temperature increase until fully dissolved (up to 55°C max). Extract solutions were stored at −20°C in the dark prior to use in the assays.

### Chemical Heme Biocrystallization Assay

**FIGURE 3:**
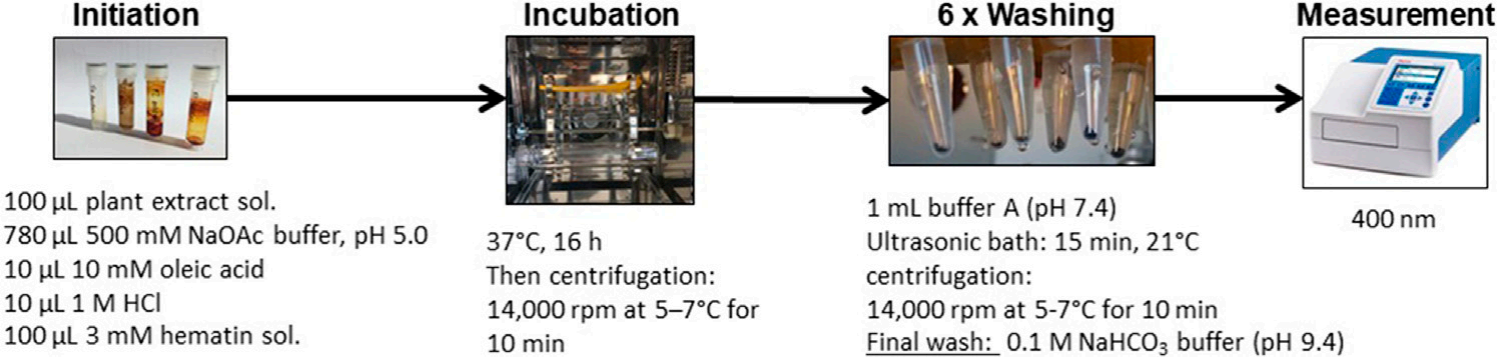
Flow scheme describing the heme biocrystallization assay procedure.

The heme biocrystallization assay was adapted from previously published studies (Baelmans et al., 2000; Heshmati Afshar et al., 2011; Sarker and Nahar, 2012), and modified. Figure 3 illustrates the modified heme biocrystallization assay. Briefly, a 3 mM hematin porcine (Sigma Aldrich) solution (freshly dissolved in 0.1 M NaOH), a 10 mM oleic acid (Carl Roth) solution, and buffer A (3.91 g/L NaCl, 0.3 g/L KCl, 0.297 g/L MgSO_4_ · 7 H_2_O, 0.901 g/L glucose, 7.1 g/L Na_2_HPO_4_, pH 5) were prepared. 100 µl of hematin solution was pipetted into each reaction tube (Eppendorf *via*l) and mixed with 10 µl 1M HCl, 10 µl 10 mM oleic acid solution, and 100 µl test sample solution. A total of 780 µl 500 mM sodium acetate buffer of pH 5 was added to each reaction tube, resulting in a total reaction volume of 1,000 µl. As a positive control, chloroquine diphosphate (Sigma Aldrich) was used. The negative control contained buffer and DMSO without test extracts. All tubes were incubated for 16 h at 37°C in a slowly rotating 360° vertical rotator setup inside an incubator. The incubation was terminated by centrifugation at 14,000 rpm at 21°C for 10 min and the supernatant was carefully removed. Next, the hemozoin pellets were repeatedly washed with 1 ml of 2.5% (*w/v*) SDS in buffer A (pH 7.4), while applying sonication (15 min, 21°C). This procedure was repeated until the supernatant was clear (usually 3–6 washes). A final wash was performed with 0.1 M sodium bicarbonate buffer (pH 9.4, Merck) and the supernatant was carefully removed. The pellets were then resuspended in 1 ml of 0.1 M NaOH. The tubes were vortexed, filtered through syringe filters, and for determination of hemozoin content, the absorbance was measured photospectrometrically at 400 nm (1 cm quartz cuvette). The % of inhibition (%I) was calculated using the following formula:

$$\%\mathrm{inhibition}=\left[\left(\mathrm{absorbanc}e_{Vehiclecontrol}-\mathrm{absorbanc}e_{Sample}\right)/\mathrm{absorbanc}e_{Vehiclecontrol}\right]\times 100$$

Data analysis and calculation of IC_50_ values was performed using Microsoft Excel^®^. All experiments were conducted in triplicate and presented as the mean values with standard deviations.

### *In vitro* Antiplasmodial Bioassay

For the assessment of antiplasmodial activity, the chloroquine-resistant *P. falciparum* K1 strain was selected. A lactate dehydrogenase (LDH) assay was used to evaluate parasite multiplication as previously described (Makler et al., 1993; Cos et al., 2006; Mesia et al., 2010). Briefly, the plasmodia were cultured in RPMI-1640 medium supplemented with 10% O^+^ human blood serum and 4% washed O^+^ human erythrocytes and maintained at 37°C under an atmosphere of 3% O_2_, 4% CO_2_, and 93% N_2_. 96-well microtiter plates were incubated for 72 h under the same incubation parameters after addition of extract solutions to the malaria parasite inoculums (1% parasitemia and 2% hematocrit). The experiments were performed in a 4-fold sample dilution series (in-test concentration of 64–16–4–1–0.25 µg/ml). After incubation, the plates were frozen and stored at −20°C. After thawing, in separate plates, 20 µl of hemolyzed parasite suspension from each thawed well was added to 100 µl Malstat^®^ reagent mixed with 10 µl of a 1/1 (*v/v*) solution of nitro blue tetrazolium (0.1 mg/ml) and phenazine ethosulfate (2 mg/ml) solutions. The plates were allowed to react in the dark for 2 h and any color change was measured photospectrometrically at 655 nm. Chloroquine diphosphate was used as a positive control. DMSO was used as solvent control. Results were expressed as % reduction in parasitemia compared to the infected controls. IC_50_ values were calculated from the dose–response curves. Each sample was tested in triplicate and experiments were repeated at least once on a separate day. Data analysis and calculation of IC_50_ values was performed using Microsoft Excel^®^.

### Cytotoxicity Against Human Lung Fibroblasts

A human diploid embryonic lung cell line (MRC-5_SV2_) was used for assessment of cytotoxicity. Tamoxifen was included as a positive control for cytotoxicity, and DMSO served as negative control. The assay procedure was previously described (McMillian et al., 2002; Mesia et al., 2010). Briefly, MRC-5_SV2_ lung fibroblasts were cultured in a minimum essential medium containing 5% FCSi, 20 mmol/L L-glutamine, and 16.5 mmol/L sodium hydrogen carbonate. A total of 190 µl of cell suspension (3 × 10^4^ MRC-5_SV2_ cells) was added to each well of a 96-well microtiter plate, containing 10 µl pre-diluted sample extracts. The experiments were performed in a 4-fold sample dilution series (in-test concentration of 64–16–4–1–0.25 µg/ml). The plate was incubated for three days at 37°C under an atmosphere of 5% CO_2_. After incubation, resazurin was added into the wells and the plate was incubated for another 4 h at 37°C. The overall cell viability or proliferation was assessed fluorimetrically at 550 nm (excitation) and 590 nm (emission). The data was processed as % reduction in cell viability in the treated cultures compared to the untreated control cultures, and CC_50_ values were calculated from the dose–response curves. The SI for antimalarial activity was calculated by dividing the CC_50_ for extract cytotoxicity by the IC_50_ for its respective antiplasmodial activity. Data analysis and calculation of IC_50_ values was performed using Microsoft Excel^®^.

### Genotoxicity Screening

To assess any potential genotoxic and mutagenic effects of plant extracts, the *Salmonella* reverse mutation test (Ames mutagenicity test), which has been described previously (Maron and Ames, 1983; Mortelmans and Zeiger, 2000; Tejs, 2008), was performed with modifications. The bacterial strains used in the assay were the two histidine-dependent *S. enterica* subsp. *enterica Typhimurium* strains TA98 and TA100. Strain IDs, characteristics, and sources are reported in Supplementary Table S2. Both strains were maintained on tryptic soy agar (TSA) after streaking from freezer stock and overnight incubation at 37°C. Overnight liquid cultures were prepared in tryptic soy broth (TSB) at 37°C and with constant shaking at 120 rpm for 16 h. The strains were checked for characteristic spontaneous revertants and reversion of its mutants caused by the positive controls (reference mutagenic substances) on each day of use. Genotypes were confirmed using the following methods: 1) R-factor: Ampicillin resistance or sensitivity was checked by spreading the *S. enterica* subsp. *enterica* wild type (DSM 11320, control), strain TA98, and strain TA100 on ampicillin-supplemented TBA plates (shown in Supplementary Figure S2); 2) *uvrB* mutation/UV sensitivity: Presence of the *uvrB* mutation in the test strains was confirmed by spreading liquid cultures in parallel stripes on TBA plates. The plates were then treated with UV light for 10 s, 30 s, 60 s, 2 min, 5 min, and 10 min, with half of each plate covered with aluminum foil; and 3) *rfa* mutation/crystal violet sensitivity: A total of 0.1 ml of an overnight culture was added to a sterile tube containing 2 ml of warm TBA, previously kept at 45°C. After vortexing for 3 s, the mixture was plated, a sterile filter paper disc (diameter: 6 mm) was placed on the agar, and 10 µl of crystal violet solution (1,000 µg/ml H_2_O) was transferred to the disc. All plates were incubated at 37°C for 24 h and subsequently checked.

The plant extract dose introduced to this assay was 500 µg/ml. Prior to use, the extract solutions were sterile filtered through 0.22 µm syringe filters (Carl Roth). A 100 µl aliquot of the test strain suspension (overnight culture), 50 µl of extract solution, 50 µl of DMSO, and 500 µl of 0.2 M phosphate buffer (pH 7.4) were added to 2.0 ml top agar (TBA containing 10% traces of biotin and histidine) and vortexed. More information is given in Supplementary Table S3. The mixture was then poured over the surface of a minimal glucose agar Petri dish and incubated at 37°C for 48 h. After incubation, the number of mutants (revertant colonies) was counted and the reversion frequency was determined as means with standard deviations. The known mutagenic substance 2-nitrofluorene (2-NF; Sigma Aldrich; 1,000 µg/ml DMSO) served as a positive control for strain TA98 and methyl methanesulfonate (MMS; Sigma Aldrich; 200 µl MMS mixed with 1.8 ml DMSO) was used for strain TA100. The final concentrations were 10 µg 2-NF/plate and 2 µl MMS/plate respectively. Each experiment was conducted in triplicate and on different days.

Absence of toxicity was assessed by observing the background bacterial growth. The mutagenicity index (MI) describes the magnitude of the mutagenic induction and was calculated by dividing the mean value of His+ revertant colonies counted on the sample treatment plates (spontaneous mutations and induced mutations) by the mean value of His+ revertant colonies on the negative control treatment plates (spontaneous mutations only). Data analysis and calculation of MI values was performed using Microsoft Excel^®^. The MI was evaluated using by a modified “twofold rule” that has previously been described (Zeiger et al., 1992; Mortelmans and Zeiger, 2000; Mathur et al., 2006). The following criteria were used for interpretation of the MI:

**Positive result (+):** An extract sample was considered a mutagen if it has a reproducible twofold increase of the reverse mutation rate (MI ≥ 2) (significantly mutagenic).
**Weakly mutagenic result (+/−):** A sample extract was considered a weak mutagen if a reproducible, close to twofold increase of the reverse mutation rate was determined (1.7 ≤ MI < 2.0).
**Negative result (−):** A sample extract was considered a non-mutagen if no increase or an increase below 170% of the reverse mutation rate was observed in at least two biological replicates (MI < 1.7).

### Human S9 Liver Fraction Treatment of Extracts

Human S9 liver fraction was incorporated into the *Salmonella* reverse mutagenicity assay to also assess the human metabolic activation (or deactivation) of potentially mutagenic plant extracts. Pooled hepatic S9 fraction represents the post-mitochondrial supernatant fraction from homogenized human liver, and S9 fractions from the same batch only were used for the experiments (Lot# SLBR5681V, S2442, Sigma Aldrich, always stored at −70°C). A sterile 0.1 M ß-nicotinamide adenine dinucleotide phosphate disodium salt (NADP, Sigma Aldrich) solution was freshly prepared and kept on ice. A 1 M glucose-6-phosphate solution and a MgCl_2_ (123 mg/ml)–KCl (81.4 mg/ml) salt solution were aseptically prepared. Subsequently, a 4% S9 mixture was freshly prepared by first mixing 69.125 ml of sterile H_2_O, 87.5 ml of sterile 0.2 M phosphate buffer (pH 7.4), 875 µl of glucose-6-phosphate solution, 3.5 ml of MgCl_2_–KCl salt solution, and 7 ml of NADP solution, and then adding 7.0 ml of pooled human S9 liver fraction. The S9 mixture was kept on ice and quickly introduced to the following steps.

Sample extracts and controls were treated with the S9 mixture *via* a pre-incubation assay. A total of 50 µl of extract solution and 50 µl DMSO were added to 500 µl S9 mixture, vortexed, and incubated at 37°C with constant shaking at 100 rpm for exactly 30 min. The mutagenic substance 2-aminofluorene (2-AF, 1 mg/ml DMSO) was used as a positive control for both *Salmonella* strains. After incubation and metabolic activation of samples, the samples were introduced to the *Salmonella* reverse mutagenicity assay by adding 2.0 ml TBA top agar and 100 µl of aliquot of the test strain suspension (overnight culture) as described in the *Genotoxicity Screening* section above. Each plate contained 20 µl human liver S9 fraction. Further information is given in Supplementary Table S4. Figure 2 was created using the biorender.com software.

## Data Availability

The original contributions presented in the study are included in the article/Supplementary Material, further inquiries can be directed to the corresponding author.
