# Supplementary material for: Pharmacological Assessment of the Antiprotozoal Activity, Cytotoxicity and Genotoxicity of Medicinal Plants Used in the Treatment of Malaria in the Greater Mpigi Region in Uganda
Source: Front Pharmacol. 2021 Jun 30;12:678535. doi: 10.3389/fphar.2021.678535 (PMC8278201; doi:10.3389/fphar.2021.678535)
Supplement: Supplementary file 1 [file DataSheet1.pdf]

Pharmacological assessment of antiprotozoal activity, cytotoxicity and genotoxicity of medicinal plants used in treatment of malaria in the Greater Mpigi Region in Uganda

Fabien Schultz[1,2*], Ogechi Favour Osuji[2], Anh Nguyen[2], Godwin Anywar[3], John R. Scheel[4,5], Guy Caljon[6], Luc Pieters[7], Leif-Alexander Garbe[2,8]

[1]Institute of Biotechnology, Faculty III - Process Sciences, Technical University of Berlin, Berlin, Germany
[2]Department of Agriculture and Food Sciences, Neubrandenburg University of Applied Sciences, Neubrandenburg, Germany
[3]Department of Plant Sciences, Microbiology and Biotechnology, Makerere University, Kampala, Uganda
[4]Department of Global Health, University of Washington, Seattle, WA, USA
[5]Department of Radiology, University of Washington, Seattle, WA, USA
[6]Laboratory of Microbiology, Parasitology and Hygiene, Faculty of Pharmaceutical, Biomedical and Veterinary Sciences, University of Antwerp, Belgium
[7]Natural Products & Food Research and Analysis (NatuRA), Department of Pharmaceutical Sciences, University of Antwerp, Belgium
[8]ZELT - Neubrandenburg Center for Nutrition and Food Technology gGmbH, Neubrandenburg, Germany

*** Correspondence:**
Fabien Schultz (Fabien.Schultz@mailbox.tu-berlin.de)

Supplementary information

Table of contents

Supplementary Table S1:
Results of the Salmonella reverse mutation assay showing the mean values of His+ revertant colonies page 3

Supplementary Figure S1:
Plate photos of revertant colonies for extract eE007 (*Albizia coriaria*, non-mutagenic against test strain TA98) and hE006 (*Solanum aculeastrum*, mutagenic against test strain TA98) page 4

Supplementary Table S2:
Information on bacterial strains used in the study page 5

Supplementary Figure S2:
Results of growth comparison of his- mutants with the wild type regarding +/- ampicillin genes page 5

Supplementary Table S3:
Overview of pipetting instructions during the Ames test procedure without metabolic activation page 6

Supplementary Table S4:
Overview of pipetting instructions during the Ames test procedure with metabolic activation (pre-treatment assay) page 6

References cited in supplementary files page 7

Supplementary Table S1:

Table S1. Results of the *Salmonella* reverse mutation assay showing the mean values of His+ revertant colonies at 500 µg/plate; nt: not tested; GI: growth inhibition

Scientific name	Extract ID	Mean values of His+ revertant colonies			
		TA98		TA100	
		Without metabolic activation	With metabolic activation	Without metabolic activation	With metabolic activation
Securidaca longipedunculata	eE001	41	447	445	926
	wE001	82	328	497	1164
	hE001	nt	186	nt	824
	mE001	36	551	369	369
	smE001	46	479	487	43
Microgramma lycopodioides	eE002	61	452	445	993
	wE002	64	527	336	721
	smE002	76	411	537	947
Ficus saussureana	eE003	47	427	316	677
	wE003	53	416	501	1011
	hE003	60	336	487	909
	mE003	45	383	428	995
	smE003	51	345	517	893
Sesamum calycinum subsp. *angustifolium*	eE004	41	397	423	984
	wE004	51	481	521	1039
	hE004	343	321	465	941
	mE004	52	560	449	1019
	smE004	36	481	488	1125
Leucas calostachys	eE005	48	276	429	735
	wE005	52	460	537	1083
	hE005	61	388	477	967
	smE005	53	417	519	988
Solanum aculeastrum	eE006	45	377	597	968
	wE006	nt	537	Nt	939
	hE006	567	288	497	852
	smE006	43	337	540	944
Albizia coriaria	eE007	39	463	416	979
	etE007	59	492	516	996
Erythrina abyssinica	eE008	44	204	328	819
	etE008	54	186	304	721
Zanthoxylum chalybeum	eE009	56	487	560	1009
	etE009	44	499	473	943
	etE017	nt	nt	nt	nt
	dietE017	37	nt	581	nt
	etE017a	46	468	445	1372
	dietE017a	39	333	385	853
Toddalia asiatica	eE010	204	435	1228	985

	etE010	94	219	963	732
	dietE010	214	319	1180	1203
	etE010a	91	509	995	889
Harungana madagascariensis	eE011	52	435	431	985
	etE011	47	320	451	1131
	dietE011	38	186	336	565
	etE011a	68	385	439	644
Morella kandtiana	etE012	45	523	471	973
	etE012a	54	564	439	848
	dietE012	52	391	220	901
Cassine buchananii	eE013	47	391	496	1053
	etE013	50	531	488	1632
	etE013a	48	445	396	919
Warburgia ugandensis	etE014a	40	437	217	1254
	dietE014	0 (GI)	0 (GI)	0 (GI)	0 (GI)
Combretum molle	eE015	46	543	352	1212
	etE015	53	573	477	955
Plectranthus hadiensis	dietE016	51	543	nt	1212
	hE016	50	268	447	821
spontaneous reverse mutations	negative control	53	496	315	983
2-NF	positive control	1000			
MMS	positive control			2064	
2-AF	positive control	54	6130	704	6001

Supplementary Figure S1:

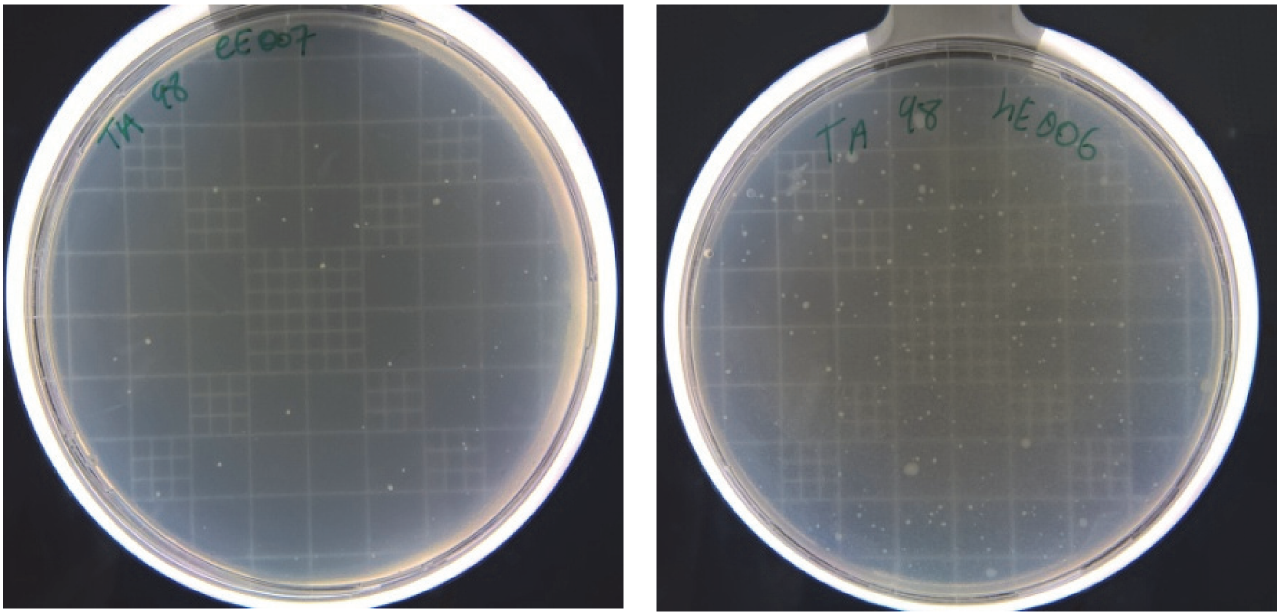

Figure S1. Plate photos of revertant colonies for extract eE007 (*Albizia coriaria*, non-mutagenic against test strain TA98) and hE006 (*Solanum aculeastrum*, mutagenic against test strain TA98)

Supplementary Table S2:

Table S2. Information on bacterial strains used in the study

Species	Strain IDs	Characteristics*	Ref.
Salmonella enterica subsp. *enterica Typhimurium*	TA 98<br>CIP 103798	Optimized for frameshift mutations, DNA target: –C–G–C–G–C–G–C–G–; his D3052 rfa Δ(gal chl bio uvrB) / pKM101<br>Source: Centre de Ressources Biologiques de l'Institut Pasteur	[1-4]
Salmonella enterica subsp. *enterica Typhimurium*	TA 100<br>CIP 103799	Optimized for base-pair substitution mutations, DNA target: –G–G–G–; hisG46 rfa Δ(gal chl bio uvrB) / pKM101<br>Source: Centre de Ressources Biologiques de l'Institut Pasteur	[3,5,6]
Salmonella enterica subsp. *enterica*	DSM-No. 11320	Wild type	[7,8]

Supplementary Figure S2:

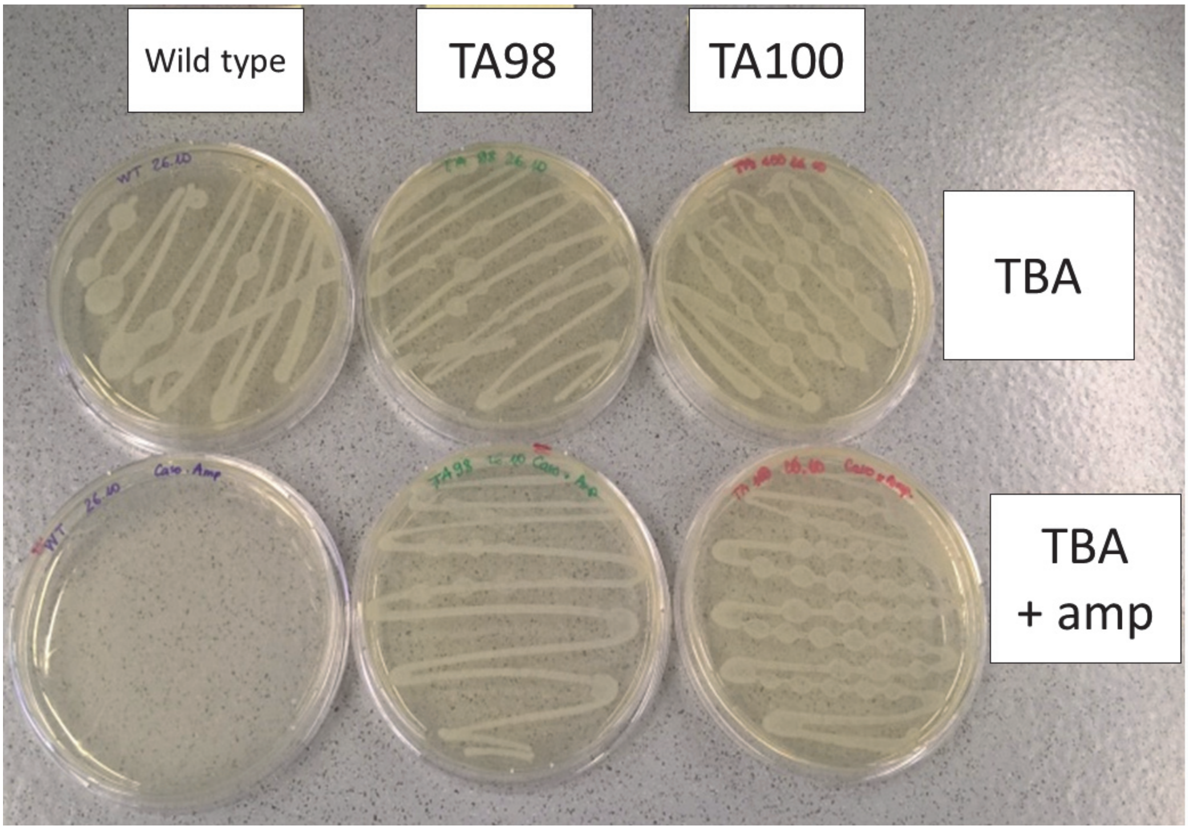

Figure S2. Results of growth comparison of his- mutants with the wild type regarding +/- ampicillin genes

Supplementary Table S3:

Table S3. Overview of pipetting instructions during the Ames test procedure without metabolic activation

	Buffer control	Sample	DMSO (vehicle control)	Positive control TA 98	Positive control TA 100
**Overnight culture (TA98 or TA100)**	100 µL	100 µL	100 µL	100 µL	100 µL
**DMSO**		50 µL	100 µL	90	90
**Buffer**	600 µL	500 µL	500 µL	500 µL	500 µL
**Plant extract solution (10 mg/ml)**		50 µL			
**MMS solution**					10 µL
**2-NF solution**				10 µL	
**Top agar**	2.0 mL	2.0 mL	2.0 mL	2.0 mL	2.0 mL

Supplementary Table S4:

Table S4. Overview of pipetting instructions during the Ames test procedure with metabolic activation

	Buffer control	Sample	DMSO (vehicle control)	Positive control TA 98	Positive control TA 100
Pre-incubation assay					
**DMSO**		50 µL	100 µL	90	90
**S9 mixture**	600 µL	500 µL	500 µL	500 µL	500 µL
**Plant extract solution (10 mg/ml)**		50 µL			
**2-AF solution**				10 µL	10 µL
Incorporation into the Ames test					
**Overnight culture (TA98 or TA100)**	100 µL	100 µL	100 µL	100 µL	100 µL
**Top agar**	2.0 mL	2.0 mL	2.0 mL	2.0 mL	2.0 mL

References cited in supplementary files

1 FBRCMi. *CIP 103799 - Salmonella enterica enterica Typhimurium*, <https://brclims.pasteur.fr/brcWeb/souche/detail/1/15636> (1993).

2 BacDive. *Salmonella enterica CIP 103798* ***BacDive ID:*** *139109*, <https://bacdive.dsmz.de/search?search=CIP+103798> (2020).

3 Mortelmans, K. & Zeiger, E. The Ames Salmonella/microsome mutagenicity assay. *Mutation Research/Fundamental and Molecular Mechanisms of Mutagenesis* **455**, 29-60, doi:https://doi.org/10.1016/S0027-5107(00)00064-6 (2000).

4 Isono, K. & Yourno, J. Chemical carcinogens as frameshift mutagens: Salmonella DNA sequence sensitive to mutagenesis by polycyclic carcinogens. *Proc. Natl. Acad. Sci. U. S. A.* **71**, 1612-1617, doi:10.1073/pnas.71.5.1612 (1974).

5 FBRCMi. *CIP 103799 Salmonella enterica enterica Typhimurium*, <https://brclims.pasteur.fr/brcWeb/souche/detail/1/15636> (1993).

6 BacDive. *Salmonella enterica CIP 103799 BacDive ID: 139108*, <https://bacdive.dsmz.de/search?search=CIP+103799> (2020).

7 DSMZ. *Salmonella enterica subsp. enterica DSM 11320*, <https://www.dsmz.de/collection/catalogue/details/culture/DSM-11320> (1996).

8 BacDive. *Salmonella enterica Zoosaloral H BacDive ID: 5113* <https://bacdive.dsmz.de/strain/5113> (2020).
